# Rheological Properties of Engine Oil with Nano-Additives Based on MoS_2_ Materials

**DOI:** 10.3390/nano12040581

**Published:** 2022-02-09

**Authors:** Łukasz Makowski, Zuzanna Bojarska, Antoni Rożeń

**Affiliations:** Faculty of Chemical and Process Engineering, Warsaw University of Technology, 00-645 Warsaw, Poland; zuzanna.bojarska.dokt@pw.edu.pl (Z.B.); antoni.rozen@pw.edu.pl (A.R.)

**Keywords:** rheological model, MoS_2_-based materials, oil nano-additives

## Abstract

To enhance oil’s tribological and rheological properties, various nano-additives are used. An example of such a nano-additive is nanosized molybdenum disulfide (MoS_2_). Due to its unique properties, MoS_2_-based materials used as lubricants have attracted significant attention. In our previous work, we developed a novel, scalable, and low-cost method for MoS_2_-based materials production using an impinging jet reactor. Hybrid nanostructures based on MoS_2_ and carbon nanomaterials (MoS_2_/CNMs) decreased the friction factor of the base oil. In the present study, a mathematical model that accounts for the viscous heating effects in rheograms was formulated. The model was used to interpret the results of rheological measurements conducted for the base oil 10W40 and its mixtures with different nanosized lubricant additives. The model of the non-isothermal Couette flow allowed us to correct the rheograms of the engine oils in the region of high shear rates where viscous heating effects become significant. The temperature correlations for the consistency and flow behavior indexes were proposed. The nanohybrid suspensions of MoS_2_ in the base oil were found to have the lowest apparent viscosity at low temperatures, typical for the cold engine startup.

## 1. Introduction

Technologies such as multi-point fuel injections, economizers, or engine control units are designed to optimize engine efficiency [1,2]. Ensuring optimal working conditions is particularly important to provide a low level of engine emissions, durability of the engine, and improvement of fuel consumption [3]. Advanced engines allow for a frequent change in engine speed. However, this necessitates the use of an oil with improved lubricating properties. One of the approaches to enhance oil properties is to use various nano-additives. Nano-additives are more effective than conventional additives due to the lower particle sedimentation, better dispersion in oil, better contact of the lubricating parts, lower pressure drop, and the fact that they do not block the fuel pump [4,5].

An example of such a nano-additive is nanosized molybdenum disulfide (MoS_2_). MoS_2_ nanolayers are bonded by weak van der Waals interactions thanks to which monolayers can easily slide in relation to each other. MoS_2_ is also characterized by good thermal stability and corrosion resistance. Due to its unique properties, MoS_2_ finds various applications on the friction material market, such as solid lubricants and additives of lubricating oils and greases [6,7,8]. In order to enhance the tribological and rheological behavior, multiple types of MoS_2_ materials have recently appeared. Among them stand out hybrid nanostructures based on MoS_2_ and carbon nanomaterials. MoS_2_ nanoparticles deposited on the carbon nanomaterial’s surface exhibit smaller particle sizes and narrower particle size distribution, resulting in better dispersion and, thus, enhanced tribological and rheological properties [9,10].

In our previous work [10] we demonstrated the influence of carbon nanomaterials (CNMs) on the tribological properties of MoS_2_ used as an additive to 10W40 oil. We compared the tribological behaviors of the base 10W40 oil to the nanosuspensions with the addition of MoS_2_ and MoS_2_/CNMs hybrid nanostructures, which proved that adding hybrid nanostructures decreased the friction factor between the engine oil and nanosuspension. Furthermore, we presented how the physicochemical properties of the nanomaterials influence the tribological and rheological behaviors of the modified engine oils. The hybrid nanostructures, MoS_2_/CNMs, were obtained through a novel method with the use of an impinging jet reactor. This method consists of the precipitation of molybdenum disulfide in an aqueous medium [11,12,13]. The presence of carbon nanomaterials in the reaction environment causes MoS_2_ nanoparticles to precipitate directly on the carbon surface. The developed surface of carbon nanomaterials also allows the obtention of smaller particle sizes and narrower particle size distribution, thus improving lubricating properties. Moreover, due to the collision of the inlet streams and a rapid change in the flow direction, the flow character changes to highly turbulent. This ensures good mixing conditions and particle precipitations on the nanoscale [14,15,16,17]. Its design, based on the constant contact of new particles of the inlet streams, results in producing particles with reproducible properties. The reactor also enables continuous and scalable production of these materials.

The viscosity of engine oil is a critical physical property that affects its ability to circulate inside an engine and effectively cool and lubricate different engine parts. Engine oil works in a broad range of conditions characterizing different engine operation modes, e.g., during a cold startup or under an excessive load. The viscosity of engine oil depends significantly on its temperature and composition [18]. The rheological properties may also be non-Newtonian and affected by additives improving oil lubricating and heat transfer properties. The viscosity variation across the lubricant film, caused by viscous heating and inefficient heat conduction in engine oils characterized by a high Prandtl number, can generate an additional load in bearings, as shown by Hunter and Zienkiewicz [19] and Snyder [20]. Measurements of the oil viscosity are often carried out at the high shear rates met in different bearing types, when viscous heating and near-wall effects or flow destabilization can falsify the results [21]. In the present study, a mathematical model that eliminates the viscous heating effects in rheograms of the power-law liquid was formulated for the stable Couette flow. The model was used to interpret the results of rheological measurements conducted by Bojarska et al. [10] for the base oil 10W40 and its mixtures with different nanosized lubricant additives.

## 2. Physicochemical Properties

The purpose of this study was to determine the mathematical model that eliminates the viscous heating effects in rheograms of 10W40 oil-based nanosuspensions. The nanosuspensions contained 1 wt.% of various additives, such as synthesized MoS_2_, reference MoS_2_ (Sigma-Aldrich, Darmstadt, Germany, <2 µm, 98%), and hybrid nanostructures (i.e., MoS_2_/GO, MoS_2_/rGO, and MoS_2_/CNTs). The additives were dispersed in 10W40 oil by ultrasonication in an ultrasonic bath for 15 min and ultrasonic homogenizer for another 15 min [10]. In order to describe the rheological properties of the base oil and nanosuspensions through mathematical equations, it is necessary to determine their density and specific heat capacity. Density measurements of the base oil and nanosuspensions were performed using an Anton Paar DMA 4500M density meter (Graz, Austria). Measurements were carried out in the temperature range of 280–350 K, with an accuracy of the measuring device of 0.05 K and density with an accuracy of 0.00005 g·cm^−3^. The experimental results were approximated by linear functions.

Specific heat capacity measurements were carried out using differential scanning calorimetry STAR 1 from Mettler Toledo (Columbus, OH, USA). Liquid nitrogen was used as the cooling agent. Measurements were performed under a protective gas atmosphere (N_2_). The experimental results were approximated by Equation (1).

(1) $$y=A+B\cdot T+C\cdot T^{2}+D\cdot T^{3}$$

where *A*, *B*, *C*, *D* are experimentally established constants for each sample in a temperature (*T* [K]) range of 278–373 K.

## 3. Model of the Non-Isothermal Couette Flow

Dissipation of mechanical energy in the Couette flow created in a rotational rheometer leads to temperature rise and changes the viscous stress and the shear rate in a liquid sample. The non-uniform temperature profile in the measuring gap between two concentric cylinders or a cone-plate system affects the viscosity of the Newtonian liquid and the flow curve of the non-Newtonian liquid. Effective removal of the dissipation heat from the stable Couette flow is challenging at high shear rates, characterizing oil flow in slits between lubricated surfaces. Thus, if the temperature of the oil sample increases by viscous heating, one has to refine the flow curve to obtain the correct apparent liquid viscosity at the predefined temperature. A classical solution of the combined Couette flow and heat transfer for temperature-dependent liquid viscosity and heat conductivity in the cone and plate system was formulated by Bird and Turian [22]. This asymptotic solution for the low Brinkman numbers was later adapted by Papathanosiou [23] to the system of concentric cylinders. In the present study, we solve equations of motion and energy for the Couette flow of the power-law liquid in the annular gap between two concentric cylinders working in the Searle configuration (rotating inner cylinder, stationary outer cylinder). The solution is obtained in the steady case when the velocity profile and the temperature profile in the measuring gap are constant and not affected by liquid acceleration and heat accumulation, i.e., during a state-of-the-art measurement of the angular velocity and torque. The velocity and temperature fields are axially symmetrical and not disturbed by Taylor vortices. It was checked that in all rheological tests the Reynolds number characterizing the flow in the measuring gap remained well below its critical value marking the formation of the three-dimensional Taylor–Couette flow [21].

The equation of motion in cylindrical coordinates [24] reads for the axially symmetrical and stable Couette flow in the annular gap between concentric cylinders as follows:

(2) $$\frac{d}{dr}\left(r^{2}\tau \right)=0$$

where r [m] is the radial coordinate, and τ [Pa] is the shear stress. Integrating Equation (2) with the boundary condition at the surface of the inner cylinder $\tau \left(r_{i}\right)=\tau_{i}$ gives:

(3) $$\tau =\frac{r_{i}^{2}\tau_{i}}{r^{2}}$$

The Ostwald–de Waele model [21] links the shear rate, $\dot{\gamma }\;\left[1/s\right]$, with the shear rate:

(4) $$\tau =k{\dot{\gamma }}^{n}$$

where the flow consistency index, k [Pa·s], and the flow behavior index, n [−], are both functions of temperature, T [K]. The shear rate is related to the tangential liquid velocity, $v_{\theta }\;\left[m/s\right]$, and to angular velocity, ω [rad/s], as follows [24]:

(5) $$\dot{\gamma }=-r\frac{d}{dr}\left(\frac{v_{\theta }}{r}\right)=-r\frac{d\omega }{dr}$$

Combining Equations (2)–(5) gives the differential equation for the angular velocity:

(6) $$\frac{d\omega }{dr}=-\frac{1}{r}{\left(\frac{r_{i}^{2}\tau_{i}}{r^{2}k}\right)}^{1/n}$$

Two non-slip conditions have to be fulfilled by the solution of Equation (6). The first condition at the surface of the rotating inner cylinder $\omega \left(r_{i}\right)=\omega_{i}$ and the second one at the surface of the stationary outer cylinder $\omega \left(r_{e}\right)=0$; two boundary conditions are required because of the unknown a priori shear stress $\tau_{i}$. When the Couette flow is isothermal, the analytical solution of Equation (6) has the following form:

(7) $$\omega =\frac{\omega_{i}r_{i}^{2/n}}{r_{e}^{2/n}-r_{i}^{2/n}}\left[{\left(\frac{r_{e}}{r}\right)}^{2/n}-1\right]$$

Introducing this result into Equations (3) and (5) gives the expressions for shear rate and shear stress:

(8) $$\dot{\gamma }=\frac{2}{n}\cdot \frac{\omega_{i}r_{i}^{2/n}}{r_{e}^{2/n}-r_{i}^{2/n}}\cdot {\left(\frac{r_{e}}{r}\right)}^{2/n}$$

(9) $$\tau =k\cdot {\left(\frac{2}{n}\cdot \frac{\omega_{i}}{r_{e}^{2/n}-r_{i}^{2/n}}\right)}^{n}{\left(\frac{r_{i}\cdot r_{e}}{r}\right)}^{2}$$

If the Couette flow is not isothermal, Equation (6) must be integrated with the energy equation. It is postulated that the viscous heat is conducted in the liquid sample in the radial direction only. In this case, the energy equation [24] can be reduced to:

(10) $$\frac{dq}{dr}=-\frac{q}{r}+\tau \cdot \dot{\gamma }$$

and the radial heat flux, $q\;\left[W/m^{2}\right]$, is given by Fourier’s law [24]:

(11) $$q=-\lambda \cdot \frac{dT}{dr}\;$$

There is no heat flux through the surface of the inner cylinder $q\left(r_{i}\right)=0$. The temperature at the surface of the outer cylinder equals $T\left(r_{e}\right)=T_{e}$. The thermal conductivity, λ [W/(m·K)], of the liquid sample depends on temperature. Numerical integration of Equations (6), (10), and (11) requires an assumption of two quantities at the surface of the inner cylinder, i.e., the shear stress $\tau_{i}$ and the liquid temperature $T_{i}$. Therefore, we applied the Levenberg–Marquardt method [25] to find $\tau_{i}$ and $T_{i}$ such that numerical integration by the fourth-order Runge–Kutta method [25] of the governing equations resulted in a liquid velocity equal to zero and a liquid temperature equal to a predefined value $T_{e}$ at the surface of the outer cylinder.

## 4. Results and Discussion

### 4.1. Physicochemical Properties

**Figure 1:**
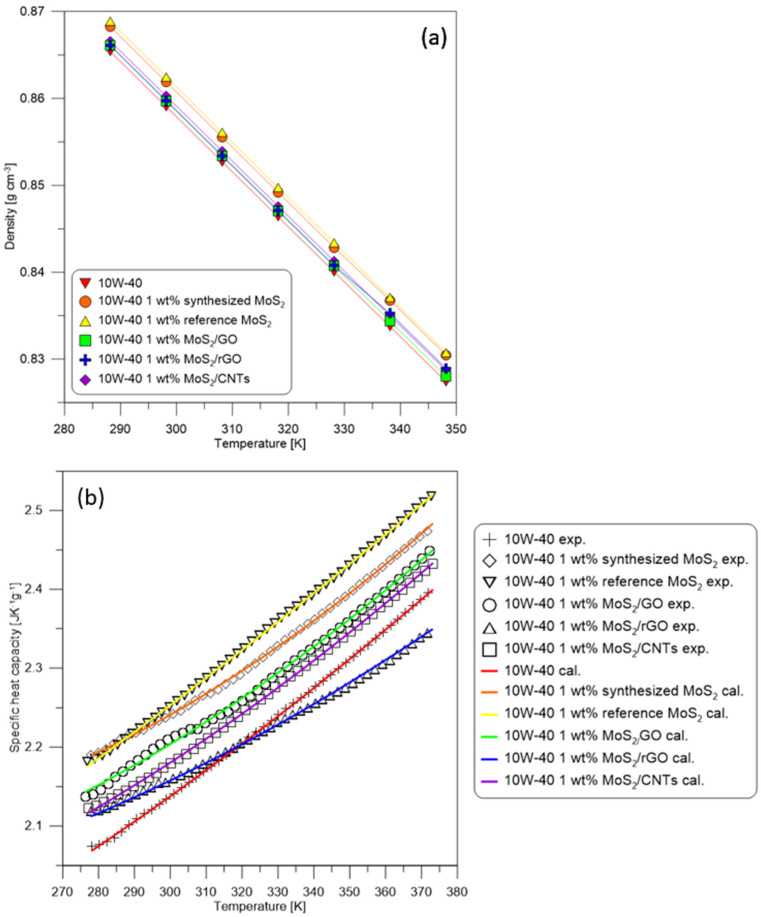
(**a**) Density and (**b**) specific heat capacity as a function of temperature of the base oil 10W40 and nanosuspensions with the addition of synthesized MoS_2_, reference MoS_2_, MoS_2_/GO, MoS_2_/rGO, and MoS_2_/CNTs.

The density of measured oils decreases linearly with increasing temperature (Figure 1a). In the case of each nanosuspension, a slight increase in density in relation to the base oil was observed. The most significant differences (up to 0.43%) were for the nanosuspension with the reference and synthesized MoS_2_, due to the higher density of pure molybdenum disulfide. A negligible increase in density (below 0.16%) is obtained for the nanosuspensions with the addition of hybrid nanostructures MoS_2_/CNMs. The carbon nanomaterials are characterized by a lower density than MoS_2_, which results in a smaller density rise of oil. The constants of the linear equation describing the density functions versus temperature with correlation coefficient (*R*^2^) are presented in Table 1.

Specific heat capacity of the measured oils increased with decreasing temperature (Figure 1b). Similar dependencies as for the density of nanosuspensions can be observed for their specific heat capacity. The additives cause an increase in specific heat capacity, and the greatest differences are obtained for the nanosuspension with addition of reference MoS_2_ (up to 5%). The experimentally established constants A–D in Equation (1) are presented in Table 1.

### 4.2. Model of the Non-Isothermal Couette Flow

**Figure 2:**
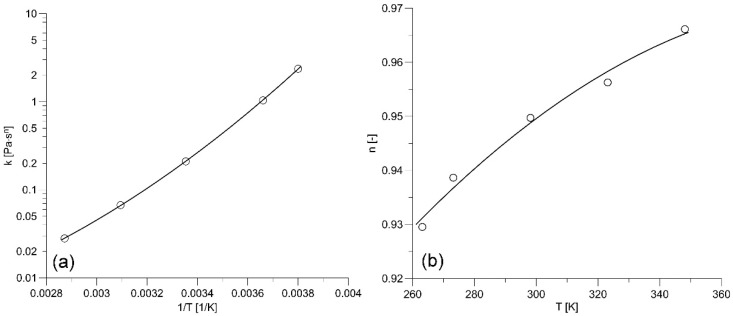
Parameters of the power model for the base oil 10W40; (o) data points, (–––) polynomial approximation: (**a**) consistency index, (**b**) flow behavior index.

Parameters of the Ostwald–de Waele model depend on temperature. Thus, correlations for the consistency and flow behavior indexes have to be determined in the low shear rate region, where viscous heating does not affect the flow curve. Analysis of the results of the rheological measurements carried out by Bojarska et al. [10] indicates that such a low shear rate region exists for all tested engine oils. Furthermore, the oil flow curve in this region can be well approximated by the power-law model—Equation (4). The measurements reported by Bojarska et al. [10] were conducted by an Anton Paar MCR 302 rheometer in the system of two concentric cylinders. The outer stationary cylinder (cup) temperature was maintained at a constant level by a Peltier module. In the low shear rate region, the shear rate and the shear stress of an oil sample at the surface of the stationary cylinder are given by the following expressions:

(12) $${\dot{\gamma }}_{e}^{+}=4\pi N\cdot \frac{r_{i}^{2/n}}{n\left(r_{e}^{2/n}-r_{i}^{2/n}\right)}$$

(13) $${\hat{\tau }}_{e}=\frac{M}{2\pi r_{e}^{2}c_{L}L}$$

where N [rev/s] is the rotation speed of the inner cylinder (rotor), and M [N·m] is the torque applied to the rotor. Equation (12) follows from Equation (8) and $\omega_{i}=2\pi N$, while Equation (13) follows from the general torque formula. The inner and outer radiuses of the measuring gap were $r_{i}=14.36\;mm$ and $r_{e}=14.46\;mm$, and the length of the measuring gap L=15.00 mm has to be corrected by $c_{L}=1.104$ to account for the gap end effects. The consistency and flow behavior indexes for all engine oils were determined in the low shear region by the least square method for temperatures ranging from −10 °C to 75 °C. Table 2 presents the power-law parameters obtained for the base engine oil 10W40. The influence of the absolute temperature on the consistency index and the flow behavior index is illustrated in Figure 2. As can be seen, the consistency index quickly increases with decreasing temperature, while the flow behavior index tends to one with increasing temperature. Second-order polynomials were applied to approximate the power-law parameters:

(14) $$\log\left(k\right)=a_{0}+a_{1}\cdot \frac{1}{T}+a_{2}{\left(\frac{1}{T}\right)}^{2}$$

(15) $$n=b_{0}+b_{1}T+b_{2}T^{2}$$

Table 3 and Table 4 present coefficients *a_i_* and *b_i_* determined for all tested engine oils by the least square method.

Sulgani and Karimipour [26] found that the thermal conductivity of 10W40 oil depends weakly on temperature, and in the present study it is approximated by the linear function

(16) $$\lambda_{10W40}=0.135-7\times 10^{-5}\left(T-298.15\right)$$

The thermal conductivity of the base liquid changes upon the addition of MoS_2_ nanoparticles. According to Shafie et al. [27], the overall thermal conductivity of such a mixture can be calculated from the Hamilton and Cross model [28]

(17) $$\lambda =\frac{\lambda_{MoS_{2}}+\left(3/\psi -1\right)\lambda_{10W40}-\left(3/\psi -1\right)\left(\lambda_{10W40}-\lambda_{MoS_{2}}\right)\phi }{\lambda_{MoS_{2}}+\left(3/\psi -1\right)\lambda_{10W40}+\phi \left(\lambda_{10W40}-\lambda_{MoS_{2}}\right)}$$

STEM images of reference and synthetic MoS_2_ nanoparticles as well as MoS_2_/GO, MoS_2_/rGO, and MoS_2_/CNTs hybrid particles presented by Bojarska et al. [10] indicate that the examined particles formed platelets. In this case, the sphericity factor ψ=0.52, whereas the thermal conductivity of MoS_2_ $\lambda_{MoS_{2}}$ equals 904.4 W/(m·K) [27]. The volume fraction of MoS_2_ nanoparticles suspended in the base oil was low (ϕ=0.0017); thus, they could not significantly increase the oil thermal conductivity.

**Figure 3:**
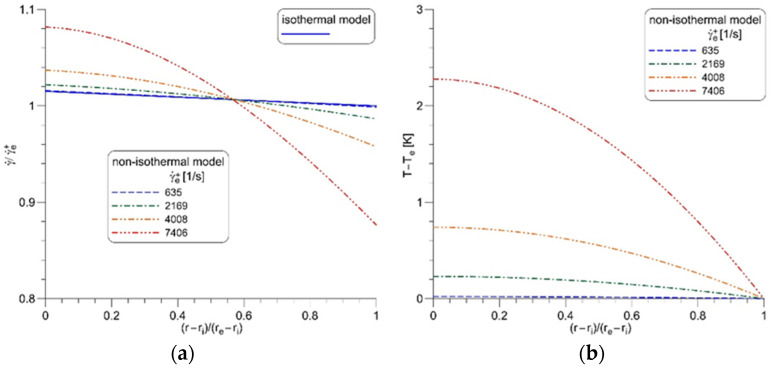
Comparison of isothermal and non-isothermal flow in the measuring gap for the base oil 10W40 at cup temperature −10 °C: (**a**) the dimensionless shear rate, (**b**) the oil temperature rise.

Determination of the consistency and flow behavior indexes in the low shear region at different temperatures using the model of isothermal Couette flow is the first step in the procedure of the rheogram correction. The next step is to apply the model of the non-isothermal Couette flow and correlations (14) and (15) to find out the extent of the high shear region. Figure 3a,b shows the shear rate and temperature profiles in the measuring gap calculated for the base oil 10W40 at the lowest cup temperature. In this case, when ${\dot{\gamma }}_{e}^{+}$ is equal to 635 1/s, the model of non-isothermal flow gives the almost exact profile of the shear rate as the isothermal flow model, and the rotor temperature is 0.02 K higher than the cup temperature. Increasing the rotational frequency of the rotor and, consequently, the shear rate in the measuring gap increases differences between predictions of both models. On the other hand, increasing the cup temperature should extend the region where the viscous heating can be neglected towards the higher shear rates.

**Figure 4:**
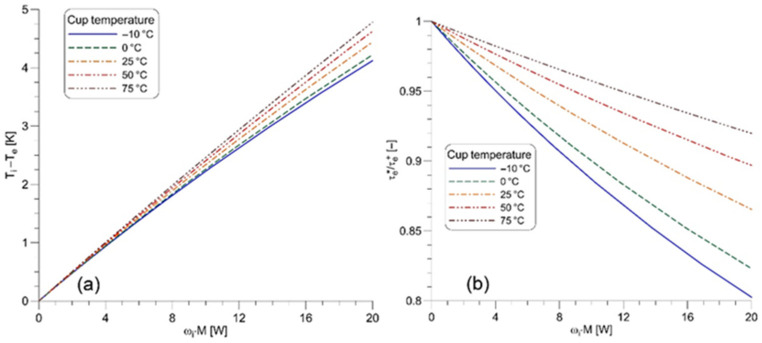
Effect of the viscous heating in the measurement of the base oil 10W40 on: (**a**) temperature difference in the measuring gap; (**b**) the shear stress at the surface of the outer cylinder.

The total heat generation rate due to viscous friction in the liquid sample is equal to the product of the angular velocity of the inner cylinder, $\omega_{i}$, and the torque applied to rotate this cylinder, M. Calculations conducted for the non-isothermal flow in the measuring system used by Bojarska et al. [10] indicate that viscous heating changes the shear stress applied to the liquid sample by 0.1% when the power input exceeds 0.1 W. One should note that the parameters of the power model, listed in Table 2, were determined for power inputs lower than 0.1 W. Figure 4a shows that the difference of the liquid temperature at the cylindrical surfaces of the measuring gap ($T_{i}-T_{e})$ gradually increases with the increasing power input. This growth is almost linear for the highest temperature of the cup (75 °C). Figure 4b illustrates the effect of power input on the ratio of the shear stress in the non-isothermal flow, $\tau_{e}^{*}$, and the shear stress in the isothermal flow

(18) $$\tau_{e}^{+}=k\cdot {\left({\dot{\gamma }}_{e}^{+}\right)}^{n}=k\cdot {\left(\frac{1}{n}\cdot \frac{4\pi N}{r_{e}^{2/n}-r_{i}^{2/n}}\right)}^{n}r_{i}^{2}$$

both calculated at the surface of the outer cylinder. Equation (18) follows directly from the power-law model given by Equations (4) and (13) for the shear rate in the isothermal flow. As expected, the shear stress predicted for the non-isothermal flow is lower than that in the isothermal flow. The most substantial effect of the power input on the ratio of shear stresses is present for the lowest cup temperature (−10 °C).

The third step in the procedure of the rheogram correction is to adjust the shear stress to eliminate the effect of viscous heating in the calculation of the apparent liquid viscosity. Such a correction is necessary for the lowest measuring temperatures when oil is very viscous and energy dissipation heating is very intensive. We adopted, in the present study, the method proposed by Štěpánek [29] and refined the flow curves to the constant cup temperature by correcting the shear stress as follows

(19) $${\tilde{\tau }}_{e}=\left(\frac{\tau_{e}^{+}}{\tau_{e}^{*}}\right)\cdot {\hat{\tau }}_{e}$$

The final step in elimination of the effect of the viscous heating is to calculate the apparent viscosity of the oil sample at the constant temperature from the expression

(20) $$\eta =\frac{{\tilde{\tau }}_{e}}{{\dot{\gamma }}_{e}^{+}}$$

**Figure 5:**
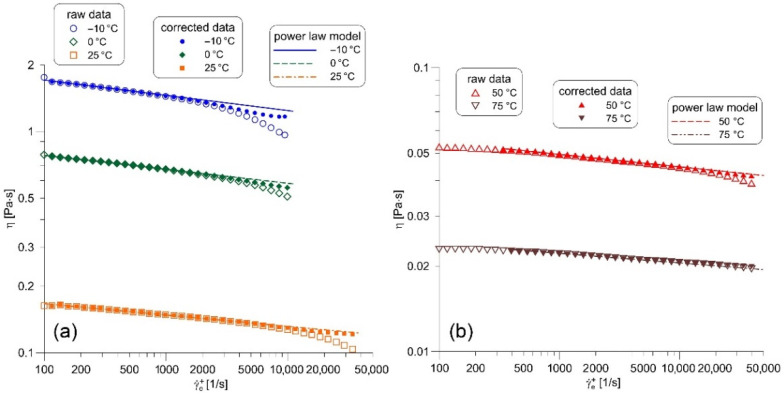
Temperature correction of the apparent viscosity of the base oil 10W40 at: (**a**) low cup temperatures; (**b**) high cup temperatures.

The apparent viscosity of the base oil 10W40 before and after correction is presented in Figure 5. The corrected viscosities are higher than the uncorrected ones, and they agree well with the viscosity curve obtained by the extrapolation of the Ostwald–de Waele model to the region of high shear rates. The viscosity refinement is considerable for the measurements conducted at a temperature lower than or equal to 50 °C. On the other hand, the viscosity correction becomes small at 75 °C. Results obtained at 50 °C and 75 °C (Figure 5b) indicate that the apparent oil viscosity changes very little in the region of the lowest shear rates (${\dot{\gamma }}_{e}^{+}<300\;s^{-1}$) where the viscosity correction is not required.

**Figure 6:**
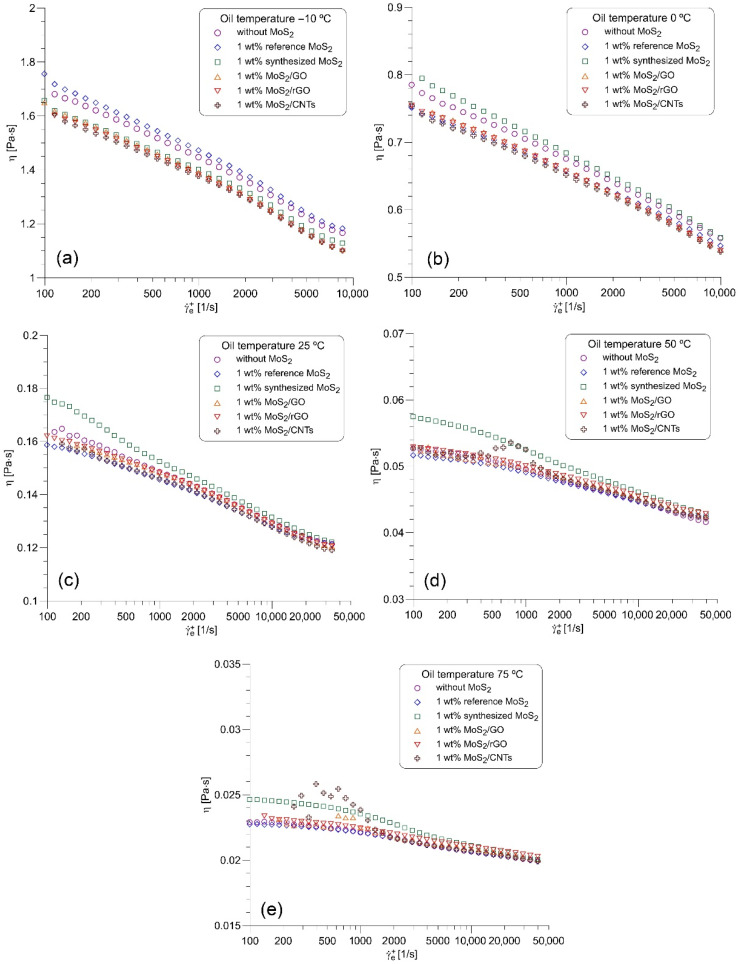
Refined viscosity curves of the pure base oil 10W40 and the base oil 10W40 with MoS_2_ nanoparticles and nanohybrid particles: (**a**) −10 °C, (**b**) 0 °C, (**c**) 25 °C, (**d**) 50 °C, (**e**) 75 °C.

The viscosity correction procedure was subsequently applied to the suspensions of MoS_2_ nanoparticles and nanohybrid particles in the base oil 10W40. Figure 6 compares the corrected viscosity of the base oil without additives with the viscosity of the base oil with different types of MoS_2_ additives. The nanohybrid suspension MoS_2_/CNTs demonstrates the lowest apparent viscosity in the entire range of shear rates at temperatures lower than and equal to 25 °C. The suspension of reference MoS_2_ nanoparticles is the most viscous in the whole range of shear rates at the lowest temperature, −10 °C. However, at higher temperatures, the suspension of synthesized MoS_2_ nanoparticles exhibits the highest apparent viscosity in the region of low and medium shear rates (${\dot{\gamma }}_{e}^{+}<3000\;s^{-1}$). In the highest shear rates region, the viscosity of the base oil and viscosities of all MoS_2_ suspensions differ very little at temperatures higher than 25 °C. The lowest viscosity at 50 °C and in the region with the highest shear rates demonstrates the base oil without MoS_2_ additives, but at 75 °C the nanohybrid suspension MoS_2_/CNTs has a slightly lower viscosity. One should also note that all nanohybrid suspensions exhibit considerable deviations of the apparent viscosity from the primary trend in the region of low and medium shear rates at 50 °C and 75 °C. These deviations can be attributed to the inhomogeneous geometry of the suspended particles of nanohybrid suspension contained in the MoS_2_/CNTs samples, as suggested by Bojarska et al. [10]. The lack of homogeneity in the shape of the particles could result in their migration in the Couette flow and in this way affect the measured torque.

## 5. Conclusions

The additives slightly increase the density and specific heat capacity of the 10W40 oil. The model of the non-isothermal Couette flow allowed us to significantly reduce viscous heating effects and correct the rheograms of the engine oils in the region of high shear rates. It was found that the Ostwald–de Waele model adequately describes the rheological properties of the tested oils in a wide range of shear rates and temperatures. The temperature correlations for the consistency and flow behavior indexes were proposed. The nanohybrid suspensions of MoS_2_ in the base oil were found to have the lowest apparent viscosity at low temperatures, typical for the cold engine startup. However, the effect of the different MoS_2_ additives on the oil’s apparent viscosity fades at high temperatures in the region of high shear rates.

## Figures and Tables

**Table 1:** Constants in the equations approximating the density (*a*, *b*) with correlation coefficient (*R*^2^) and specific heat capacity (*A*, *B*, *C*, *D*) as a function of temperature of nanosuspensions.

Engine Oil						
	Without MoS_2_	1 wt.% Reference MoS_2_	1 wt.% Synthesized MoS_2_	1 wt.% MoS_2_/GO	1 wt.% MoS_2_/rGO	1 wt.% MoS_2_/CNTs
*a*	−6.3286 × 10^−4^	−6.3482 × 10^−4^	−6.2968 × 10^−4^	−6.3375 × 10^−4^	−6.1825 × 10^−4^	−6.3189 × 10^−4^
*b*	1.0477	1.0518	1.0496	1.0487	1.0440	1.0487
*R* ^2^	0.999999	0.999995	0.999959	0.999996	0.999665	0.999998
*A*	1.5617	1.4982	2.3823	2.1193	2.0400	1.9094
*B*	5.9018 × 10^−4^	1.6111 × 10^−3^	−3.5117 × 10^−3^	−2.1927 × 10^−3^	−1.4000 × 10^−3^	−1.1446 × 10^−3^
*C*	4.4437 × 10^−6^	3.0218 × 10^−6^	1.0141 × 10^−5^	8.2476 × 10^−6^	5.9712 × 10^−6^	6.8309 × 10^−6^
*D*	0	0	0	0	0	0

**Table 2:** Consistency index and flow behavior index of 10W40 engine oil at different temperatures.

Temperature [°C]	*k* [Pa·s^n^]	*n* [/]	Correlation Coefficient
−10	2.363	0.930	0.9997
0	1.036	0.939	0.9991
25	0.2100	0.950	0.9997
50	0.06695	0.956	0.9999
75	0.02804	0.966	0.9995

**Table 3:** Polynomial coefficients in Equation (13) for the oil consistency index.

Engine Oil 10W40	*a*_0_ [/]	*a*_1_ [K]	*a*_2_ [K^2^]	Correlation Coefficient
without MoS_2_	−1.250	−1.747 × 10^3^	5.720 × 10^5^	1.0000
1 wt.% reference MoS_2_	−0.6319	−2.170 × 10^3^	6.400 × 10^5^	0.9998
1 wt.% synthesized MoS_2_	−2.343	−9.517 × 10^2^	4.369 × 10^5^	1.0000
1 wt.% MoS_2_/GO	−0.6348	−2.146 × 10^3^	6.338 × 10^5^	1.0000
1 wt.% MoS_2_/rGO	−0.7899	−2.031 × 10^3^	6.144 × 10^5^	1.0000
1 wt.% MoS_2_/CNTs	−1.994	−1.272 × 10^3^	4.976 × 10^5^	1.0000

**Table 4:** Polynomial coefficients in Equation (14) for the oil flow behavior index.

Engine Oil 10W40	*b*_0_ [/]	*b*_1_ [1/K]	*b*_2_ [1/K^2^]	Correlation Coefficient
without MoS_2_	0.6386	1.649 × 10^−3^	−2.041 × 10^−6^	0.9853
1 wt.% reference MoS_2_	0.5347	2.285 × 10^−3^	−2.917 × 10^−6^	0.9827
1 wt.% synthesized MoS_2_	1.0872	−1.145 × 10^−3^	2.142 × 10^−6^	0.9947
1 wt.% MoS_2_/GO	−0.1944	4.504 × 10^−3^	−6.528 × 10^−6^	0.9851
1 wt.% MoS_2_/rGO	0.2720	3.991 × 10^−3^	−5.714 × 10^−6^	0.9913
1 wt.% MoS_2_/CNTs	0.7343	9.526 × 10^−4^	−8.431 × 10^−7^	0.9986

## Data Availability

Data presented in this article is available on request from the corresponding author.
